# The Effect of Oxidative Stress on the Chicken Ovary: Involvement of Microbiota and Melatonin Interventions

**DOI:** 10.3390/antiox10091422

**Published:** 2021-09-06

**Authors:** Jianping Wang, Ru Jia, Haojie Gong, Pietro Celi, Yong Zhuo, Xuemei Ding, Shiping Bai, Qiufeng Zeng, Huadong Yin, Shengyu Xu, Jingbo Liu, Xiangbing Mao, Keying Zhang

**Affiliations:** 1 Animal Nutrition Institute, Sichuan Agricultural University, Chengdu 611130, China; rujia@sicau.edu.cn (R.J.); haojiegong@sicau.edu.cn (H.G.); zhuoyong@sicau.edu.cn (Y.Z.); dingxuemei@sicau.edu.cn (X.D.); shipingbai@sicau.edu.cn (S.B.); zqf@sicau.edu.cn (Q.Z.); yinhuadong@sicau.edu.cn (H.Y.); shengyuxu@sicau.edu.cn (S.X.); 13856@sicau.edu.cn (X.M.); zkeying@sicau.edu.cn (K.Z.); 2 Faculty of Veterinary and Agricultural Sciences, The University of Melbourne, Parkville 3010, Australia; pietro.celi@adisseo.com; 3 School of Life Science and Engineering, Southwest University of Science and Technology, Mianyang 621010, China; liuswust@163.com

**Keywords:** follicle atresia, cecal microbiota, metabolomics, melatonin, SIRT1-P53/FoxO1 pathway, ovary stress biomarker

## Abstract

The poultry ovary is used as a classic model to study ovarian biology and ovarian cancer. Stress factors induced oxidative stress to cause follicle atresia, which may be a fundamental reason for the reduction in fertility in older laying hens or in aging women. In the present study, we set out to characterize the relationships between oxidative stress and ovarian function. Layers (62 weeks of age; BW = 1.42 ± 0.12 kg) were injected with tert-butyl hydroperoxide (tBHP) at 0 (CON) and 800 μmol/kg BW (oxidative stress group, OS) for 24 days and the role of melatonin (Mel) on tBHP-induced ovary oxidative stress was assessed through ovary culture in vitro. The OS (800 μmol/kg BW tert-butyl hydroperoxide) treatment decreased the reproduction performance and ovarian follicle numbers. OS decreased the expression of SIRT1 and increased the *P53* and *FoxO1* expression of the ovary. A decreased Firmicutes to Bacteroidetes ratio, enriched *Marinifilaceae* (family), *Odoribacter* (genus) and *Bacteroides_plebeius* (species) were observed in the cecum of the OS group. Using Mel in vitro enhanced the follicle numbers and decreased the ovary cell apoptosis induced by tBHP. In addition, it increased the expression of SIRT1 and decreased the P53 and FoxO1 expression. These findings indicated that oxidative stress could decrease the laying performance, ovarian function and influence gut microbiota and body metabolites in the layer model, while the melatonin exerts an amelioration the ovary oxidative stress through SIRT1-P53/FoxO1 pathway.

## 1. Introduction

In recent years, there has been a growing interest in the role of reactive oxygen species (ROS) and oxidative stress in female reproduction [1,2]. Oxidative stress refers to elevated intracellular levels of ROS derived from cellular metabolism or environmental stimuli that cause peroxidation of unsaturated lipids in cell membranes and oxidation of proteins and DNA, leading to further damage of the cell integrity and normal functions [3,4]. Oxidative stress has been proven to be linked to the internal mechanism for aging [5,6], many environmental stressors and chemical toxicants (gamma radiation, mycotoxins, heavy metals, pesticides, etc.), and health disorders [7,8,9,10]. Moreover, a large number of studies have shown that an excessive increase in ROS production will induce rapid primordial follicle loss and follicular atresia to lead to reproductive dysfunction [11,12,13,14]. However, the underlying pathological and molecular mechanisms in oxidative stress-induced fertility deterioration remain unexplored.

Recent studies in mammals have found that oxidative stress affected nutrient metabolism, altering the body’s homeostasis and exerting detrimental effects on the gut microbiota and intestinal function [15,16]. In addition, it has been proved that gut microbial dysbiosis is closely related to inflammation, diseases, and other stress disorders [17,18]. However, until now, the underlying changes in microbiota and their relationship with reproductive function under oxidative stress conditions had not yet been elucidated.

Melatonin (Mel, N-acetyl-5-methoxytryptamine), is an indoleamine, can be mainly bio-synthesized in the pineal gland and the initial precursor of melatonin biosynthesis is tryptophan [19,20]. Mel is also synthesized in numerous peripheral organs, including the intestine, retina, skin and harderian gland [20,21]. It has been shown that Mel reduces oxidative stress by scavenging pro-oxidative molecules such as superoxide anions and detoxifying oxygen and nitrogen-based toxic reactant [21,22]. Melatonin has been shown to have direct effects on ovarian function and microbiota [6,18,19]. Melatonin is identified in human preovulatory follicular fluid, where its concentration is higher than that in peripheral serum [20]. Previous studies have been suggested that Mel may enhance follicle growth by increasing levels of antioxidant enzymes and reproductive hormones in laying hens [22,23]. These findings suggest that Mel is related to the reproductive process, but the underlying mechanism is unclear.

As an NAD+-dependent protein deacetylase, silent information regulator 1 (SIRT1) is involved in the deacetylation of histones and transcriptional factors regulating the cell cycle, and has been a principal modulator of metabolism and resistance to oxidative stress [24,25,26]. SIRT1 was found to be ubiquitously expressed in the ovaries of animals, and has also been involved in the regulation of ovarian aging, follicular development, and oocyte maturation [24,27]. Previous research has shown that SIRT1 is involved in the protective effects of melatonin [28]; however, the exact mechanism is not elucidated. Foxo1 (forkhead box O1), a member of the FOXO transcription factor family, is an important player in regulating cell fate and combating oxidative stress and a downstream target of SIRT1 [29,30]. Is the SIRT1-FoxO1 pathway involved in the antioxidative stress function of melatonin in the ovary?

The poultry ovary is a classic model for studying ovarian biology, follicular development and ovarian cancer. In this study, we hypothesized that oxidative stress would decrease ovarian function by changing body metabolism and gut microbiota, while Mel administration would prevent oxidative damage and maintain the ovarian function of laying hens. Thus, this study aimed to investigate the negative effect of oxidative stress on ovary function, gut microbiota and serum metabolome in a layer model. We also determined the modulating effect of melatonin on ovarian follicle atresia in order to identify the relationship between melatonin and oxidative stress-induced ovarian dysfunction in vitro.

## 2. Materials and Methods

### 2.1. Animals, Diets and Design

**Figure 1:**
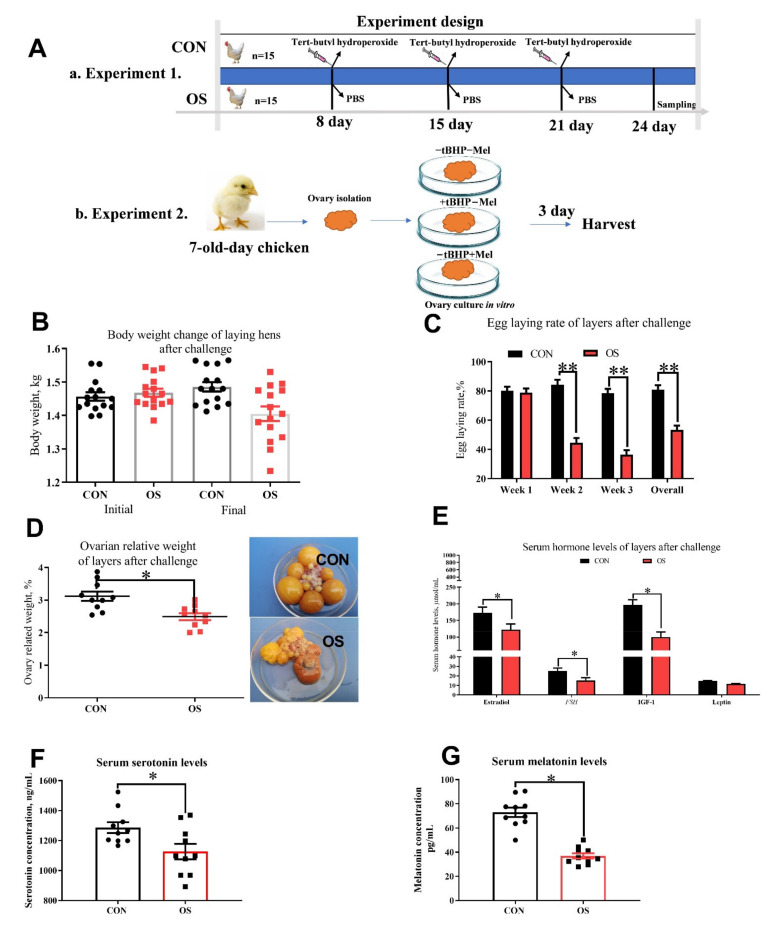
Oxidative stress (induced by tBHP) reduced egg-laying rate, ovary indices and serum hormone levels (Experiment 1). Data are means ± SEM represented by vertical bars or plot individual values ± SEM. (**A**) Schematic illustration of the experimental design. In Experiment 1, layers were fed the same basal diet for 24 days and with the tBHP (OS) or PBS (CON) injection at 9 AM of the d 8, 15, and 21. In Experiment 2, the ovaries of 7-day-old chickens are isolated for in vitro culture, and received different culture medium with 0 tBHP + 0 melatonin (−tBHP − Mel), 50 μmol/mL tBHP (+tBHP − Mel), and 50 μmol/mL tBHP + 100 ng/mL melatonin (+tBHP + Mel). (**B**) Body weight. (**C**) Egg-laying rate after challenge. (**D**) Ovarian relative weight. (**E**) Serum reproductive and metabolism-related hormone levels. (**F**) Serum serotonin levels, and (**G**) Serum melatonin levels. FSH = follicle-stimulating hormone, IGF-1 = insulin-like growth factor-1, CON = same dosage of PBS, OS = 800 μmol/kg BW of tBHP. Statistical significance was evaluated by *t*-test, * *p* < 0.05, ** *p* < 0.01.

Thirty Lohmann laying hens (62 week of age; BW = 1.42 ± 0.12 kg) were randomly allocated into two experimental groups (*n* = 15). Layers were received an intraperitoneal injection of 5 mL of 0 (CON, phosphate-buffered saline) or 800 μmol/kg BW (oxidative stress group, OS; the dosage were determined according to Kučera et al. 2014) tert-butyl hydroperoxide (tBHP) at 9 a.m. on the 8th, 15th and 22nd day of the 24-day experiment. The experiment protocol is depicted in Figure 1(Aa). All hens were housed in an environmentally controlled room (45–60% relative humidity, 22 ± 2 °C temperature; lighting cycle, 16 h/day; 05:00 a.m. to 09:00 p.m. for light). Hens were supplied with water ad libitum and fed the same amount (100 g/kg) of complete feeding mixture in mash form (the diet nutrient composition is shown in Supplementary Materials Table S1).

### 2.2. Sample Collection and Measurements

Oviposition time of the laying hens was recorded, and blood samples of each hen were collected immediately following the first oviposition in the series according to the method described by Ren [31]. Serum samples were obtained from these blood samples by incubation at 4 °C for 30 min and subsequent centrifugation at 1500× *g* for 20 min. The same hens were then sacrificed with an overdose intravenous injection of sodium pentobarbital (300 mg/kg BW), the ovary was immediately harvested after washing with phosphate-buffered saline (PBS; pH = 7.2–7.4) and placed into 4% paraformaldehyde (pH = 7.2) fixation and paraffin for ovarian follicle counts (H&E) (described in 2.6) and cell apoptosis (TUNEL) analysis. The cecum contents were carefully collected, immediately placed in cryogenic vials, stored immediately at −20 °C in a portable freezer, delivered to the laboratory and stored at −80 °C until DNA extraction.

### 2.3. Culture of Layer Ovaries In Vitro

Ovaries organ from 7-day-old chickens were used to evaluate the effect of Mel on oxidative stress-induced follicle atresia. Ovary culture was performed in vitro as previously described [32,33]. Ovary were cultured in graded level of melatonin and tBHP (−tBHP − Mel = 0 Mel + 0 tBHP; +tBHP − Mel = 50 μmol/mL+ 0 Mel: +tBHP + Mel = 50 μmol/mL+ 100 pg/mL) according to the previous study and also the melatonin levels in ovarian tissue. The in vitro experiment procedure is depicted in Figure 1(Ab).

### 2.4. Reproduction Performance and Blood Hormone Assay

Egg-laying rates were recorded every day and the body weight of the layer was recorded on the onset and end day of the experiment (*n* = 15). On day 24 of the animal trial, blood samples were collected (*n* = 10) from the wing after 12 h of fasting and the serum were separated by incubation at 4 °C for 30 min and subsequent centrifugation at 1500× *g* for 20 min. Commercial enzyme-linked immunosorbent assay (ELISA) kits were used to detect the serum concentration of estrogen (estradiol, ABIN5524298), follicle-stimulating hormone (FSH, ELISAGenie, CHFI00020, London, UK), insulin-like growth factor-1 (IGF-1, ELISAGenie, CHFI00088), leptin (ELISAGenie, CHEB0024), melatonin (IBL, #RE54021, Germany) and serotonin (Abcam, ab133053, Cambridge, UK).

### 2.5. Tissue Antioxidant Capacity

The activities of the following enzymes (*n* = 10) [superoxide dismutase (SOD), glutathione s-transferase (GST)], Cu-ZnSOD and MnSOD the levels of protein carbonyl (PC), total antioxidant capacity (T-AOC) and glutathione (GSH) and malondialdehyde (MDA) in ovarian tissue were determined by colorimetric enzymatic assays, and serum MDA concentration was measured by chemical colorimetric method, using an ELISA microplate reader (Tecan Co., Grodingen, Austria) and assay kits (T-SOD, A001-1-2; A004-1-1; Cu-ZnSOD, A001-4-1; GST, A004-1-1; PC, A087-1-2; T-AOC, A015-1-2; GSH, A005-1-2; MDA, A003-1), which were purchased from Nanjing Jiancheng Bioengineering Institute of China. All assays were conducted and interpreted according to the manufacturer’s manual without any modification.

### 2.6. Histology and Follicle Counts

The quantification of ovarian follicles (*n* = 10) was performed as previously described with small modifications [29,30]. In brief, paraffin-embedded ovaries were serially sectioned at 5-μm thickness and stained with H&E for morphological observation. Every fifth section was counted for both ovaries in vivo experiment and in vitro cultured trial. Different classes of ovarian follicles were defined as previously described [22,34]. Those with oocytes surrounded by one layer of flattened five to eight somatic cells were defined as primordial follicles. Primary follicles consisted of an oocyte surrounded with one layer containing one enlarged cell or a whole layer of cuboidal pre-granulosa cells. Prehierarchical follicles contained more than one layer of granulosa cells, including small white follicles (SWFs, 2–4 mm), large white follicles (LWFs, 4–6 mm) and small yellow follicles (SYFs, 6–8 mm). Atretic follicles were defined as previously described [34,35]: early-aretic follicles were traversed by few or no blood vessels, and the granulosa cell layer had become either partially or completely detached from the basement membrane; the surface of the progressed-atretic follicles was opaque, the granulosa cell layer was totally disorganized within the follicle.

### 2.7. TUNEL Assay

At the end of experiment, the ovary tissue (*n* = 10) was quickly removed and placed immediately into methylaldehyde; then a TUNEL assay was performed, using an In Situ Cell Apoptosis Detection Kit I (POD), according to the manufacturer’s protocol (Roche Group, Switzerland). Using BA200Digital (Mike Audi Industrial Group Co., Ltd., Xiamen, China) for image acquisition. The diaminobenzidine reacted with the labeled sample to generate an insoluble brown (light green in Experiment 2) signal, while blue-green to greenish tan signified nonapoptotic cells. Overall, 100 images were taken to measure cell apoptosis, and apoptosis rate was defined as the percentage of apoptotic cells (granulosa cells) in 100 granulosa cells counted. Observations were conducted in four different directional areas of each follicle. If the positive cells in the four areas of a follicle exceeded one-third, the follicle was defined as atretic. The percentage of atretic follicles (less than 2 mm in diameter) per section was calculated as the number of atretic follicles.

### 2.8. Ovary Function Related mRNA Expression by Real-Time PCR

The total RNA and real-time RT-PCR were carried out as described previously [9]. In brief, total RNA were extracted with Trizol followed by DNase1 treatment to remove genomic DNA. Gene expression of caspase 3, caspase 9, Bcl-2 (B-cell lymphoma-2), Bax, SIRT1 (silent information regulator 1), FoxO1 (forkhead box O1), and P53 was determined by quantitative real-time PCR in the ovary of layers by ABI 7900 Real-Time PCR system (ABI Biotechnology, Eldersburg, MD, USA). The primer information for all the genes is listed in Supplementary Materials Table S2. Each sample was assayed in triplicate and β–actin was used as the house-keeper genes. The 2^−ΔΔCT^ method was used to calculate target gene expression, and mRNA expression in CON was used as baseline relative to treatment groups (i.e., fold-change).

### 2.9. Western Blotting Assay

Total protein expression in ovarian tissues (*n* = 10; Experiment 2) were detected by Western blotting as previously described [36,37]. Primary antibodies against FoxO1 (#2880; 1:1000 dilution; 78–82KD; Cell Signaling Technology), and P53 (#29453; 1:1000 dilution; 53KD; Novus), SIRT1 (#8469;1:1000 dilution; 110KD; Cell Signaling Technology) and β-actin (#4970S) were obtained and a pre-study was performed to confirm its specificity. The goat anti-rabbit IgG-HRP (1:10,000 dilution; Santa Cruz) was used as the secondary antibody. Meanwhile, a mouse monoclonal antibody against β-actin (1:5000 dilution; ImmunoWay, Plano, TX, USA) acted as a reference, and the goat anti-mouse IgG-HRP (1:10,000 dilution; Santa Cruz) was used as the secondary antibody. Bound antibodies were detected by the ECL Prime Western blotting detection reagent (GE-Healthcare) using ImageJ software (National Institutes of Health, Bethesda, MD, USA).

### 2.10. Gut Microbiota and Short-Chain Fatty Acids (SCFA) Analysis

The composition of the microbial community in the cecum digesta (*n* = 10) was assessed with high-throughput pyrosequencing as recently described [37]; the sequencing and bioinformatics analysis were performed by Novogene Bioinformatics Technology Co. (Tianjin, China). SCFA (*n* = 10) including acetate, propionate and butyrate in the cecum content, were also analyzed using Agilent 6890 gas chromatograph (Agilent Technologies, Santa Clara, CA, USA) following previous protocols [38].

### 2.11. Metabolic Profiling Analysis

The ovarian tissue sample (*n* = 4) from laying hens (2 ovarian tissue samples from the same treatment were pooled together to test the metabolic profiling, but 1 pooled sample from replicate 9 and 10 were contaminated so only 4 pooled sample were used here) were taken to analyze the effect of oxidative stress on ovarian metabolic and biochemical alternation in layers. The method was performed by Novogene Bioinformatics Technology Co. (Tianjin, China) according to the previous published (see the Supplementary Materials files). Differences were indicated when *p*-value was <0.05, VIP (Variable Importance in the Projection) > 1, and only fold changes >1.5 were considered.

### 2.12. Statistical Analysis

Data were analyzed by one-way analysis of variance (ANOVA) using GLM procedure of SAS 9.2 (SAS Institute, Cary, NC, USA) and GraphPad Prism (GraphPad Inc., La Jolla, CA, USA), and the difference between two treatments was performed by student *t*-test (Experiment 1) and Tukey’s test (Experiment 2). The results are presented as mean ± SEM.

## 3. Results

### 3.1. Oxidative Stress Reduced Reproductive Performance, Ovary Indices and Serum Hormone Concentration

The hens in the OS group presented lower egg-laying rate throughout the trial compared to the CON group; moreover, the ovary indices were lower in hens challenged with tBHP (Figure 1C,D; *p* < 0.05). A decrease in the serum concentration of estradiol, FSH, IGF-1, serotonin and melatonin were observed in the OS group compared to the CON one (Figure 1E–G; *p* < 0.05). No differences in body weight were noted between the CON and OS groups (Figure 1B; *p* > 0.05).

### 3.2. tBHP Induced Oxidative Stress Decreased Ovarian Function

**Figure 2:**
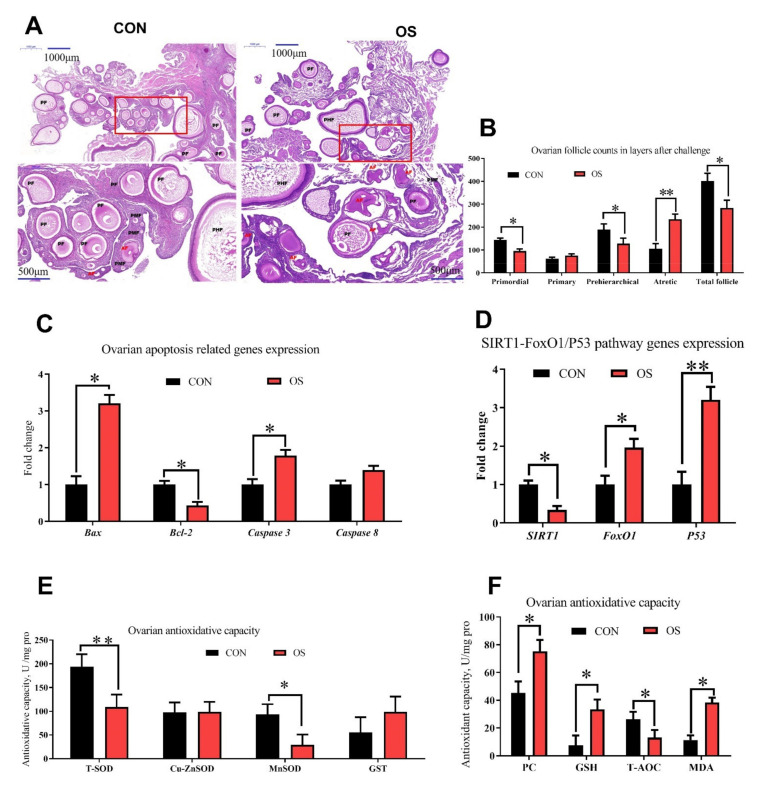
Oxidative stress (induced by tBHP) decreased ovary function (Experiment 1). (**A**,**B**) Ovarian histology of layers after 24 days treatment and follicle numbers at each developmental stage (PMF = primordial follicle; PF = primary follicle; PHF = prehierarchical follicle; AF = atretic follicle). (**C**,**D**) RT-PCR analysis for mRNA expression levels related to ovarian follicular apoptosis and antioxidant related gene expression. (**E**,**F**) Antioxidant capacity analysis for ovary with antioxidant enzyme activities and oxidation product. Data are means ± SEM represented by vertical bars or plot individual values (*n* = 10) ± SEM. CON = same dosage of PBS, OS = 800 μmol/kg BW of tBHP, Bcl-2 = B-cell lymphoma-2, SIRT1 = sirtuin 1, FoxO1 = forkhead box O1, T-SOD = total superoxide dismutase, GST = glutathione S transferase, PC = protein carbonyl, GSH = glutathione, T-AOC = total antioxidant capacity, MDA = malondialdehyde. Statistical significance was evaluated by *t*-test, * *p* < 0.05, ** *p* < 0.01.

**Figure 3:**
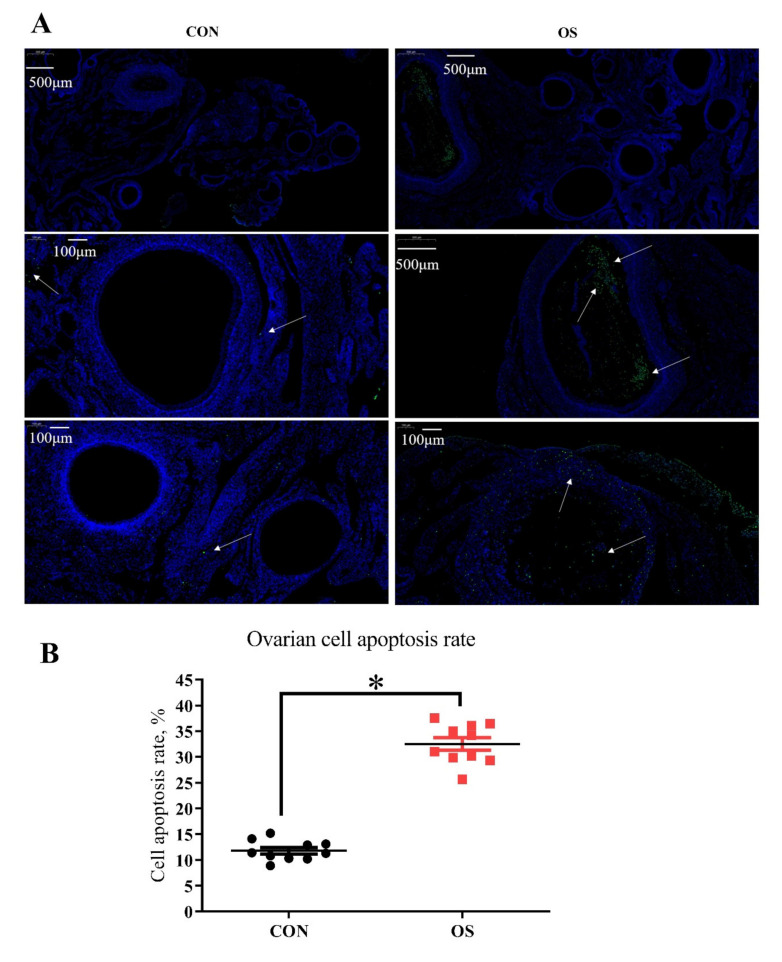
Oxidative stress (induced by tBHP) increased the cell apoptosis rate of ovary (Experiment 1). (**A**) TUNEL analysis for cell apoptosis in ovary; (**B**) The immunofluorescence results of TUNEL with the green color presents the positive cells. Data are plot individual values (*n* = 10). CON = same dosage of PBS, OS = 800 μmol/kg BW of tBHP. Statistical significance was evaluated by *t*-test, * *p* < 0.05.

Compared to the CON group, layers challenged with tBHP had lower numbers of primordial, prehierarchical and total follicles (Figure 2A,B; *p* < 0.05). The number of atretic follicles and the ovary cell apoptosis rate were higher in the OS groups than in the CON ones (Figure 2A,B and Figure 3A,B; *p* < 0.05). The levels of antioxidant parameters (T-SOD, MnSOD, T-AOC) were decreased while concentration of oxidant products (PC, MDA) and GSH (intracellular antioxidant) was higher in the OS treatment (Figure 2E,F; *p* < 0.05), suggesting that the tBHP challenge induced oxidative stress in the ovary. The real-time PCR results indicated that the mRNA expression level related to ovarian follicular apoptosis factors (Bax, caspase 3, caspase 8, FoxO1, and P53) was greater, while the mRNA abundance of anti-apoptotic genes (Bcl-2 and SIRT1) was lower in the OS group compared to the CON group (Figure 2C,D; *p* < 0.05).

### 3.3. Oxidative Stress Reduced Cecal Short Chain Fatty Acids and Induced Gut Microbiota Dysbiosis

**Figure 4:**
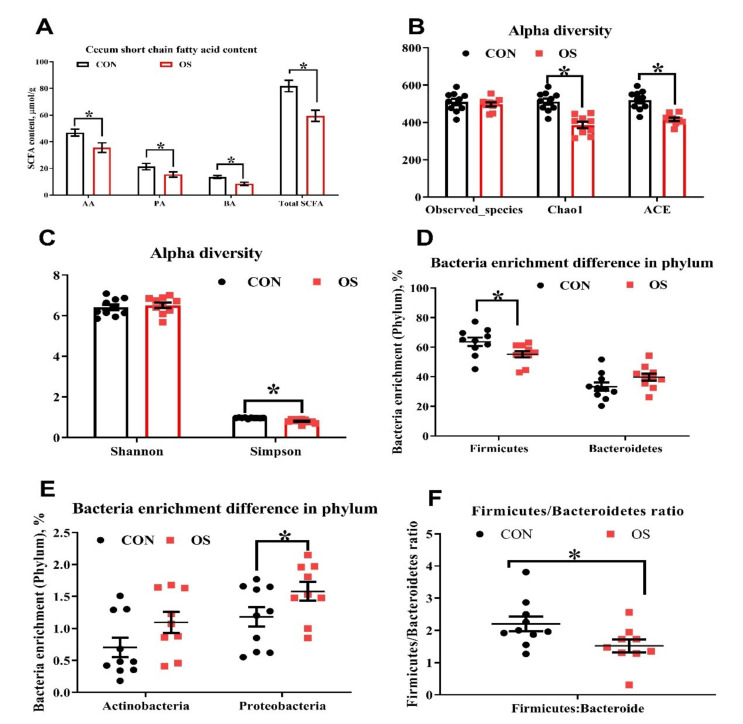
Oxidative stress (induced by tBHP) reduced cecal short chain fatty acid concentration (Experiment 1). Data are means ±SEM represented by vertical bars or plot individual values. (**A**) Short chain fatty acid concentration in cecal digesta. (**B**,**C**) Alpha diversity of cecum microbiota, with Observed species, Chao 1, ACE (**B**) and Shannon and Simpson index (**C**). (**D**–**F**). The difference *n* = 10. Statistical significance was evaluated by the Student’s *t*-test, * *p* < 0.05.

The concentration of the main short chain fatty acids (acetic, propionic and butyric) and the total SCFA in the cecum contents of layers treated with tBHP were lower compared to those in the CON group (Figure 4A; *p* < 0.05). As suggested by the decrease in the ACE, Chao1, and Simpson indexes relative to CON group, the tBHP challenge resulted in reduced microbial diversity (Figure 4B,C; *p* < 0.05). Structural changes in intestinal microbiota were assessed by PCoA based on the unweighted UniFrac metric, which indicated that layers in both groups had obvious clustering (Figure 4D).

**Figure 5:**
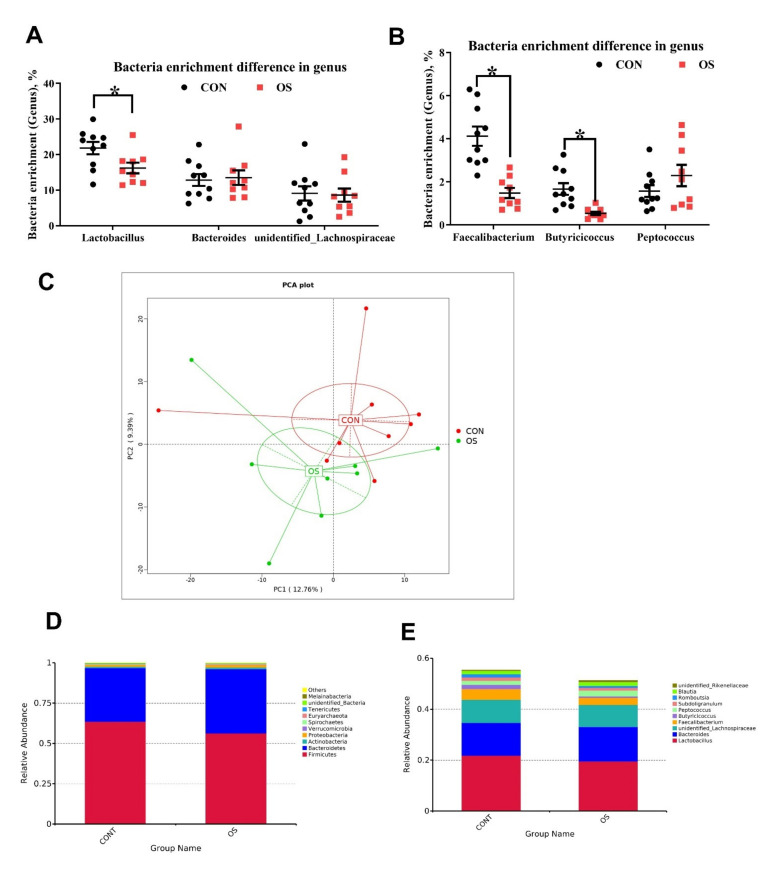
Oxidative stress (induced by tBHP) changed microbiota diversity (Experiment 1). Data are means ± SEM represented by vertical bars or plot individual values. (**A**,**B**) The bacteria abundance enrichment difference in the genus. (**C**) The principal coordinate analysis (PCoA) of the cecum microbiota based on unweighted UniFrac metrc. (**D**,**E**) The relative abundance of the top 10 phylum (**D**) and genus (**E**). The difference *n* = 10. Statistical significance was evaluated by the Student’s *t*-test, * *p* < 0.05.

**Figure 6:**
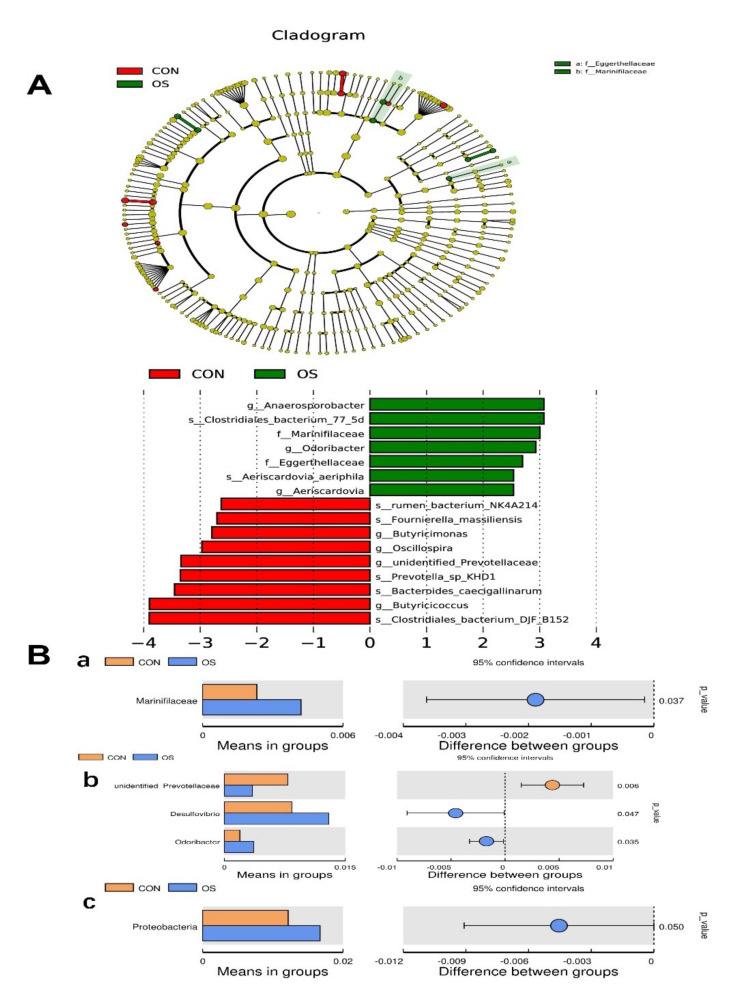
Oxidative stress (induced by tBHP) changed microbiota enrichment (Experiment 1). Data are means ±SEM represented by vertical bars or plot individual values. (**A**) Linear discrimination analysis coupled with effect size (LEfSe) identified most differentially abundant taxa in the cecum with LDA significant threshold > 3 were shown. (Red) CON enriched taxa; (Green) OS enriched taxa. (**B**) Analysis of different species between groups at different levels. (**a**) Family, (**b**) Genus, (**c**) Species. The difference *n* = 10. Statistical significance was evaluated by the Student’s *t*-test.

As analyzed by Adonis and LEfSe (log10 LDA > 3), the tBHP challenge significantly reduced the relative abundance of Firmicutes (Phylum), *Lactobacillus* (genus), *Faecalibacterium* (genus), *unidentified_Prevotellaceae* (genus), *Prevotella_sp_KHD1* (Species), *Butyricicoccus* (Species), and *Clostridiales_bacterium_DJF-B152* (species) (Figure 4E,F; Figure 5A–E; Figure 6; *p* < 0.05), while it led to an increase in the enrichment of *Proteobacteria* (Phylum), *Marinifilaceae* (family), *Odoribacter* (genus) and *Clostridiales_bacterium_77_5d* (species). The *Firmicutes* to *Bacteroidetes* ratio was also lower in the OS group compared with the CON group (Figure 6; *p* < 0.05).

### 3.4. Oxidative Stress Changed Amino Acid Biosynthesis and Ovarian Serotonin and Melatonin Concentration

**Figure 7:**
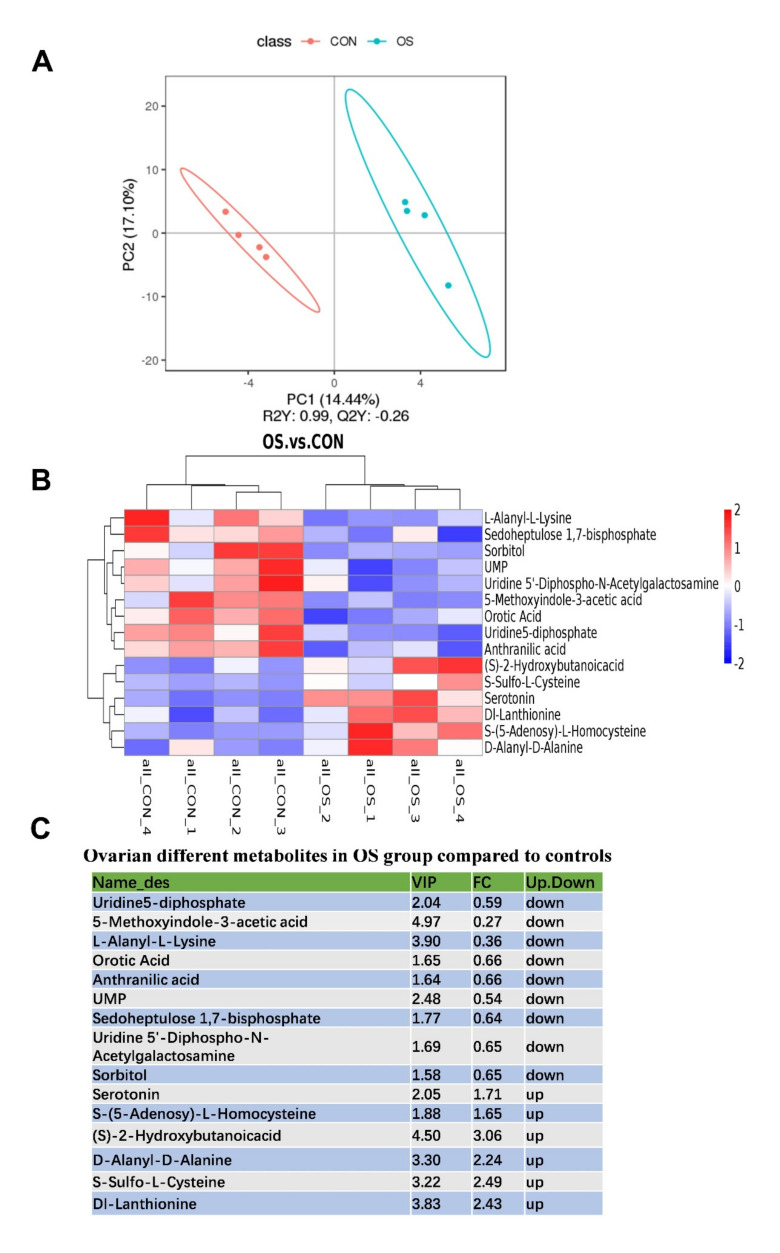
Oxidative stress (induced by tBHP) changed the serum metabolites. (**A**) The principal component analysis (PCA) of the serum metabolites. (**B**) Heatmap of the different serum metabolites. (**C**) Ovarian different metabolites in OS group compared to controls. *p* < 0.05.

**Figure 8:**
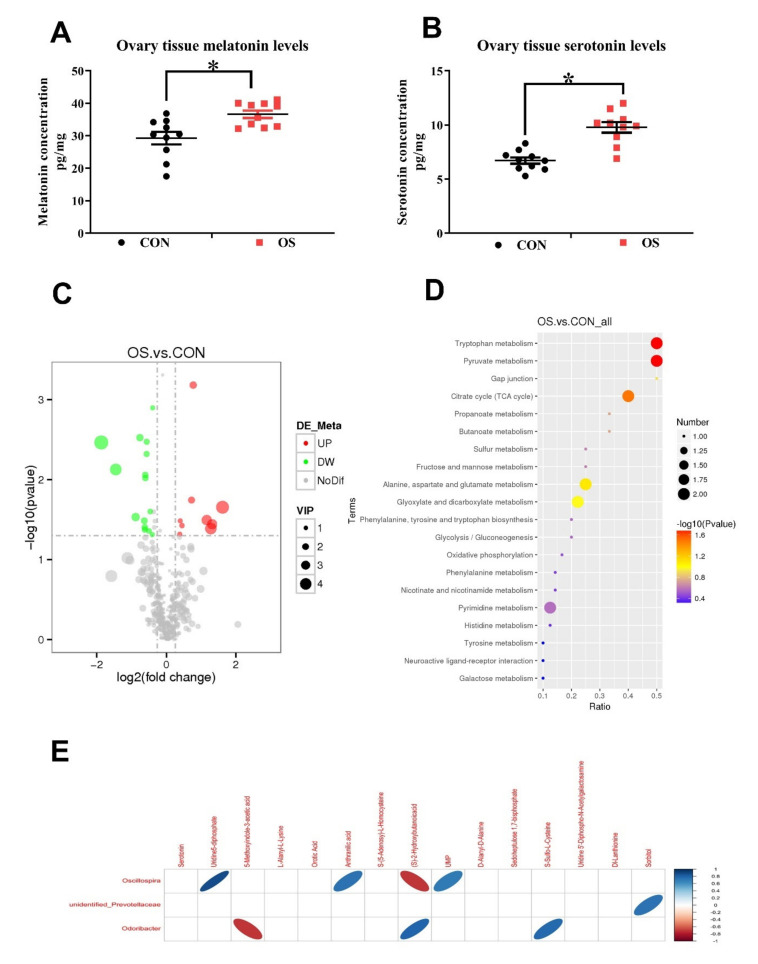
Oxidative stress (induced by tBHP) reduced increased serotonin and melatonin levels in the ovary and changed body amino acid biosynthesis and metabolism. (**A**) Ovarian tissue melatonin levels. (**B**) Ovarian tissue serotonin levels. (**C**) Description of the different metabolites between OS and CON group by volcano plot. (**D**) KEGG pathway enrichment of target metabolites. (**E**) Spearsman Correlationship between metabolites and microbiota. Statistical significance was evaluated by the Student’s *t*-test, * *p* < 0.05.

In this study, a total of 481 metabolites were identified; the layer in the OS group showed a notable metabolite alteration as compared to the CON group, as showed by PLS-DA analysis (Figure 7A). We observed that 30 metabolites were altered between the OS and CON groups, of which 21 of them were downregulated, and the rest 9 were upregulated (Figure 7B,C; *p* < 0.05, VIP > 1). The significantly altered metabolites were distributed as amino acids, lipids, organic acids, nucleosides, sugar alcohol, aliphatic acyclic compounds, aromatic heteropolycyclic compounds, and aliphatic heteromonocyclic compounds. In order to identify possible pathways relevant to the development of oxidative stress, all of the attributed metabolites were subjected to a high-quality KEGG metabolic pathways database (Figure 7C). As shown in Figure 8D, the concentration of differential metabolites in the corresponding metabolic pathways of Tryptophan metabolism, Pyruvate metabolism, Gap junction and Citrate cycle (TCA cycle) (*p* < 0.05). The concentrations of serotonin and melatonin in the ovary of layers challenged with tBHP were increased (Figure 8A,B; *p* < 0.05), which was similar to the observed pattern of metabolomic profile (Figure 7B,C; *p* < 0.05, VIP > 1).

Correlations between metabolites and microbiota with significant differences between the two groups were obtained via Spearman’s correlation analysis. As shown in Figure 8E, we observed that the bacteria genera, including *Oscillospira, unidentified_Prevotellaceae* and *Odoribacter* were most closely related to the changed metabolites in the ovary of OS group (*p* < 0.05). In particular, bacteria of the *Odoribacter* genera were negatively correlated to the concentration of S-Sulfo-L-Cysteine and positively correlated to the 5-Methoxyindole-3-acetic acid (indole and its derivatives), while bacteria of the Oscillospira genera were related mostly to the organic acids (anthranilic acid, (S)-2-Hydroxybutanoicacid) and nucleotide metabolism process (UMP, uridine5-diphosphate).

### 3.5. Melatonin Ameliorates tBHP Induced Oxidative Stress Ovarian Dysfunction In Vitro

**Figure 9:**
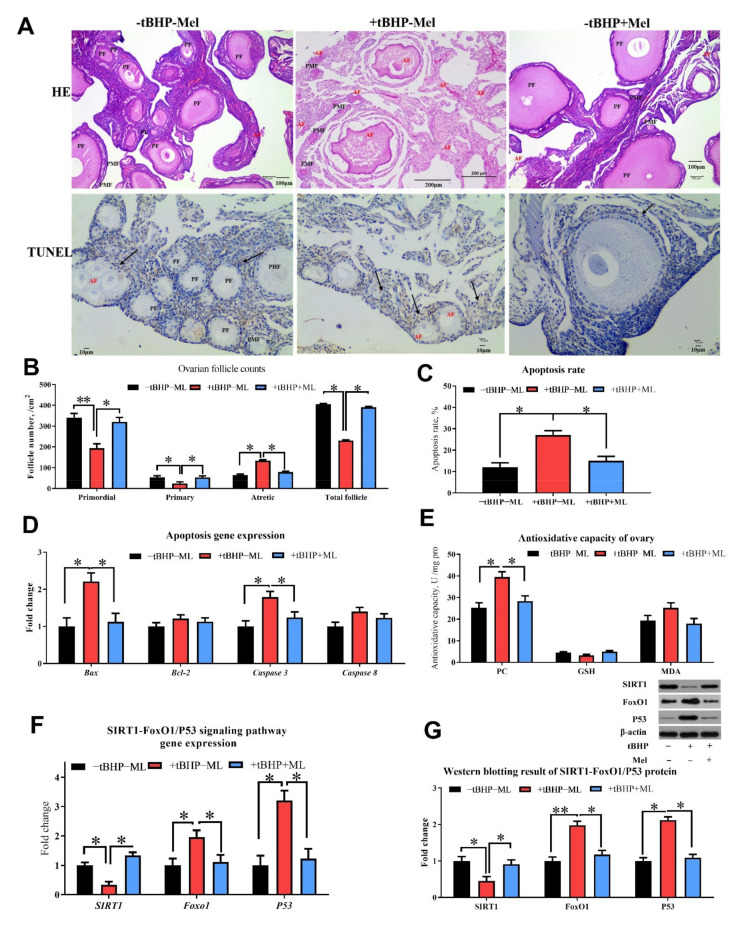
Melatonin ameliorates oxidative stress (tBHP) induced ovarian dysfunction in vitro (Experiment 2). (**A**,**B**) Ovarian histology and TUNEL analysis of layers after three days of treatment and follicle counts at each developmental stage (PMF = primordial follicle; PF = primary follicle; AF = atretic follicle). TUNEL analysis for cell apoptosis in ovary (**A**,**C**) showed the TUNEL results with the brown color presents the positive cells. (**D**) RT-PCR analysis for mRNA expression levels related to ovarian apoptosis gene expression. (**E**) Antioxidant capacity analysis for ovary with oxidation product. (**F**,**G**) the western blotting result of apoptosis associate protein (Bax, Bcl-2, caspase 3 and cleaved-caspase 3). Data are shown as averages and error bars represent SEM± SEM; statistically significant differences are shown with different letters (*p* < 0.05) with one-way ANOVA with Tukey’s test. −tBHP − Mel = 0 tBHP + 0 melatonin, +tBHP − Mel = 50 μmol/mL tBHP, +tBHP + Mel = 50 μmol/mL tBHP + 100 ng/mL melatonin, Bcl-2 = B-cell lymphoma-2, SIRT1 = silent information regulator 1, FoxO1 = forkhead box O1, PC = protein carbonyl, GSH = glutathione, MDA = malondialdehyde. Statistical significance was evaluated by *t*-test, * *p* < 0.05, ** *p* < 0.01.

Seven-day-old chicken ovaries were cultured and treated with tBHP and Mel for 3 days in vitro, and the histological analysis revealed that the addition of tBHP in the cell culture medium decreased the number of primordial follicles, primary and total follicles and increased the number of atretic follicles, cell apoptosis rate, and also the proapoptotic markers (Bax and caspase 3) in the ovary, whereas the addition of Mel was able to reverse the detrimental effect of tBHP (Figure 8A–D; *p* < 0.05). The concentration of protein oxidant products (PC) was higher in the ovary treated with tBHP, while it decreased after Mel administration (Figure 8E; *p* < 0.05). *SIRT1* gene expression and protein levels were decreased, while *FoxO1* and *P53* were increased in the OS group; the addition of Mel was able to reverse this effect (Figure 9F,G; *p* < 0.05).

## 4. Discussion

Proper functioning of the ovary is critical to maintaining fertility and overall health, and ovarian function depends on the maintenance and normal development of ovarian follicles [39,40]. Accelerated metabolism occurs in rapidly proliferating granulosa cells (GCs) within developing follicles, leading to increased ROS production. Moreover, accumulating evidence demonstrates that excessive ROS are the key signals in the initiation of apoptosis in granulosa cells of both primordial and growing follicles by diverse stimuli, such as alcohol, radiation, and smoking, as well as malnutrition and obesity [14,41,42]. Melatonin, an endogenous component of follicular fluid, has been suggested to improve GCs resistance to oxidative stress in vitro cell model [43,44,45]. In this study, we investigated the role of Mel in protecting ovarian follicles from atresia via the SIRT1-FoxO1/P53 pathway; we also examined the role of the gut microbiota in this process.

Oxidative stress induces apoptosis and affects cellular homeostasis, which results from dysregulation between antioxidant and pro-oxidant availability [42]. PC and MDA are produced during protein and lipid peroxidation, and they can be indicative of OS [46]. The markedly lower levels of T-SOD, Mn-SOD, T-AOC activities, along with significantly elevated MDA and PC levels within the ovary of tBHP challenged layers (OS group) suggest the presence of oxidative damage and also indicates that our model was successfully established in the current study.

We observed that OS decreased the layers’ reproductive performance as indicated by the lower egg-laying rate, decreased hormone levels (lower estradiol, FSH, IGF-1), and also by the smaller primordial follicle reserve and increased atresia in the ovaries of the tBHP challenged layers. In our previous study, we also found that oxidative stress (induced by high levels of molybdenum and vanadium) decreased egg production in layers and the addition of antioxidants (tea polyphenols) was able to reverse this effect by improving the antioxidant capacity and gut microbiota balance [9,47]. Studies in mammals have found that oxidative stress can reduce the number of follicles in each stage of the ovarian cycle and impair ovarian function [48,49,50,51,52]. Additionally, previous studies have also reported that oxidative stress decreases hormone secretion (including lower estradiol, FSH, LH and IGF-1) and impairs the glutathione redox cycle [50,53]. Recent animal studies indicate that IGF-1 exerts antioxidant effects and anti-inflammatory effects in animals [54,55]. Leptin was demonstrated to exert an important role in the regulation of ovarian folliculogenesis indirectly via control of luteinizing hormone and FSH secretion [52]; however, the OS did not affect leptin levels, but decreased LH and FSH levels in the current study.

Interestingly, both melatonin and serotonin serum levels were decreased after the OS challenge, but the levels of melatonin and serotonin levels were enhanced in the ovary, while the addition of melatonin in the in vitro experiment was able to reverse these adverse effects induced by the tBHP challenge. To date, there is a paucity of data elucidating the mechanism contributing to the gut–ovary axis in the literature. In the present study, we aimed to establish a link between the gastrointestinal microbiome and ovarian function. In the current study, we found that the microbial diversity was reduced by the tBHP challenge. In agreement with our observations, another study also reported that mice fed a high-fat diet resulted in oxidative stress and the increased ROS levels disrupted the intestinal microenvironment, ultimately resulting in dysbiosis [56,57].

In this study, we observed that Firmicutes and Bacteroidetes were the most abundant at the phylum level, and Bacteroides and Lactobacillus were the dominant genera in all dietary treatments. The Firmicutes to Bacteroidetes ratio is an important biomarker of gastrointestinal functionality and can be used as an indicator of eubiosis conditions in the gastrointestinal tract [58]. Previous studies have also demonstrated that the gut microbiota of obese subjects (humans) is characterized by a lower abundance of Firmicutes and a higher abundance of Bacteroidetes compared to their lean counterparts [51]. At the same time, it has been reported that oxidative stress has significantly increased the estimators of richness and community diversity of the gut microbiota of sows [57]. In addition, other studies have demonstrated that during conditions of intestinal dysbiosis, an excessive bioavailability of ROS molecules can contribute to an increase in oxidative stress [59,60]. It could be argued that these discrepancies may be related to the form of the type of animal or the level of oxidative stress. 

In this study, although the cecal microbiota of the OS group was slightly similar to that of the CON one, the abundance of *Marinifilaceae, Odoribacter* and *Bacteroides_plebeius* in the OS group was significantly increased. Bacteroidetes are plant polysaccharide degraders and propionate producers that can improve intestinal barrier function and reduce inflammation and oxidative stress [46,61]. It has been reported that the relative abundance of Bacteroides is enriched in the gut of the host treated with antioxidants [62]. Our observation is in agreement with that of Wang [63], who found that oxidative stress significantly increases the relative abundance of *Bacteroides_f_Bacteroidaceae* in the gut of sows.

In this study, we observed that tryptophan metabolism, pyruvate metabolism, and the TCA cycle were disrupted by the tBHP challenge. While serotonin was upregulated, anthranilic acid, succinic acid, and oxaloacetate were downregulated. Tryptophan is an essential amino acid and is generally considered as the second-limiting amino acid in the most based diets of layers. Tryptophan metabolized in animals comes from two sources: one is an endogenous amino acid that is broken down by tissue protein, and the other is the exogenous amino acid that is digested and absorbed from the diet [64]. It has been reported that oxidative stress significantly decreased tryptophan/large neutral amino acids and serotonin concentrations in pigs, suggesting that oxidative stress might increase tryptophan metabolism [65]. This is consistent with our research results. 

The tryptophan metabolism has been associated with various nutrients such as carbohydrates, proteins during the metabolic process [66]. An increase in the metabolism of tryptophan during oxidative stress could affect other physiological processes. On the other hand, tryptophan is also the precursor of Mel, and which can also be synthesized in the ovary [22]. Therefore, the decreasing levels of tryptophan in serum may correspond to the higher Mel and serotonin in the ovary. Pyruvate can realize the mutual conversion of carbohydrates, lipids, and amino acids in the body through the acetyl CoA and tricarboxylic acid cycles [67]. Therefore, pyruvate plays an important pivotal role in the metabolic connection of the three major nutrients. Moreover, the increased metabolism of pyruvate in cases of oxidative stress may be related to the increased need for oxygen and energy during the production of ROS.

It has been shown that supplementing exogenous pyruvate can prevent oxidative tissue stress [68]; however, in this study, we observed that oxidative stress promoted pyruvate metabolism. This apparent discrepancy may be due to different experimental models and different tissues tested, which needs to be clarified in further investigations. Oxidative stress could affect the levels of metabolites from glycolysis and the TCA cycle and production of adenine nucleotides [69]. In addition, it has been shown that an increase in oxidative stress and a decrease in the TCA cycle enzymes activity may cause the distal peripheral nerve to rely on truncated TCA cycle metabolism in rats [70]. Tryptophan catabolism has been recognized as an important player in inflammation and immune response. In this study, we found that oxidative stress caused a decrease in succinic acid and oxaloacetate in the TCA, which is consistent with previous studies. Oxidative stress will hinder the TCA and pyruvate metabolism, and ultimately affect the metabolism of carbohydrates, lipids, and amino acids.

SIRT1 is an NAD+-dependent protein deacetylase, and it has been proved to be involved in the protective effects of melatonin [71]. In this study, we found that melatonin levels were decreased in oxidative stress and the exogenous addition of Mel could mitigate the negative impact of oxidative stress and improve ovarian functionality in tBHP challenged layers. It has been shown that SIRT1 exerts its beneficial effects via the reduction of oxidative stress and endoplasmic reticulum stress in mitochondria [28,72]. Our results also indicated that the SIRT1 was increased by melatonin addition in vitro and decreased FoxO1 and P53 expression. As reported previously, melatonin was able to protect mouse granulosa cells against oxidative damage by inhibiting FoxO1-mediated autophagy [72,73]. 

The animal model we used (ovaries from laying hens) may be different from the ovaries of productive mammalian animals. There are distinct histological and physiological differences according to the reproductive stage of the animal. Moreover, “a link between the gastrointestinal microbiome and ovarian function” could be speculated in the current animal model, but cannot be established for other species and humans especially. However, the literature about oxidative stress on the ovary function of poultry is not well elucidated, which needs to be explored in future studies.

## 5. Conclusions

In conclusion, we found that oxidative stress could decrease laying performance, ovarian function and induce gut microbiota dysbiosis and disrupt serum metabolites in tBHP challenged layers; melatonin was able to reverse the impact of oxidative stress at the ovarian level through the SIRT1-P53/FoxO1 pathway (Graphical Abstract).

## Data Availability

The data presented in this study are available in this article and also Supplementary Material here.

## Supplementary Materials

The following are available online at https://www.mdpi.com/article/10.3390/antiox10091422/s1, Table S1: Composition and nutrient levels of basal diet, Table S2: Related gene and primer information.
