# Supplementary material for: The Effect of Oxidative Stress on the Chicken Ovary: Involvement of Microbiota and Melatonin Interventions
Source: Antioxidants (Basel). 2021 Sep 6;10(9):1422. doi: 10.3390/antiox10091422 (PMC8472688; doi:10.3390/antiox10091422)
Supplement: Supplementary file 1 [file antioxidants-10-01422-s001.zip › antioxidants-1309058-supplementary.pdf]

Table S1. Composition and nutrient levels of basal diet

Item, %	Amount
Corn	57.72
Wheat bran	5.00
Soybean oil	2.00
Soybean meal	23.30
$CaCO_3$	9.70
Calcium hydrophosphate	1.05
NaCl	0.40
L-Lysine·HCl	0.10
DL-Methionine	0.10
Tryptophan	0.05
Threonine	0.05
Choline chloride	0.10
Vitamin premix[1]	0.03
Mineral premix[2]	0.40
Total	100.00
Analyzed nutrient levels, %	
ME, kcal/kg	2650.00
Crude protein	15.50
Calcium	4.00
Total phosphorus	0.53
Lysine	0.77
Methionine	0.36
Methionine + cysteine	0.58

[1]Provided per kilogram of diet: vitamin A, 8000 IU; vitamin B_1, 0.4 mg; vitamin B_2, 1.2 mg; D-pantothenate, 5 mg; vitamin B_6, 7.55 mg; vitamin B_{12}, 6 µg; vitamin D_3 2000 IU; vitamin E 5 IU, vitamin K_3 1 mg; biotin, 4 mg; niacin acid 7 mg; folic acid 100 µg.

[2]Provided per kilogram of diets: Cu (as copper sulfate)：8 mg, Fe (as ferrous sulfate) 60 mg，Mn (as manganese sulfate) 60 mg，Zn (as zinc sulfate) 80 mg，I (as potassium iodide) 1 mg，Se (as sodium selenite) 0.30 mg.

[3]Calculated according to NRC (1994).

Table S2. Related gene and primer information

Genes	Orientation	Primer Sequences (5'-3')	Product size	Accession number
Caspase 3	Forward	AAAGATGGACCACGCTCAGG	204	NM_204725
	Reverse	TGAACGAGATGACAGTCCGG		
Caspase 9	Forward	TATGGTGGAGGACATGCAGA	99	XM_424580.5
	Reverse	AATATTGGGAAGGCCTGCTT		
Bcl-2	Forward	ACCATGAATGAAACCGTGCC	181	NM_205339.2
	Reverse	TTGTCGTAGCCTCTTCTCCC		
Bax	Forward	GTACGTCAATGTGGTCACCC	210	XM_015274882
	Reverse	TGGGATAATGCTGGGGTTGA		
SIRT1	Forward	TAGCCAATGGTTTCCACTCC	149	NM_001004767.1
	Reverse	AAGAATTGTCCGTGGGTCTG		
FoxO1	Forward	AAGAGCGTGCCCTACTTCAA	125	NM_204328.1
	Reverse	TTCCCTGTTCCCTCATTCTG		
P53	Forward	TACTCCCCGGTGCTGAATAA	134	NM_205264.1
SIRT1	Forward	TAGCCAATGGTTTCCACTCC	149	NM_001004767.1
β-actin	Forward	GCTACAGCTTCACCACCACA	90	NM_205518.1
	Reverse	TCTCCTGCTCGAAATCCAGT		
